# Evaluating the Usefulness of Online Tools for Detecting Inappropriate Prescriptions in the Geriatric Population at a Tertiary Care Teaching Hospital in Central India: A Retrospective Study

**DOI:** 10.7759/cureus.77254

**Published:** 2025-01-10

**Authors:** Yazhini Rajendran, Yogendra Keche, Nitin R Gaikwad, Suryaprakash Dhaneria

**Affiliations:** 1 Pharmacology, All India Institute of Medical Sciences, Raipur, Raipur, IND; 2 Pharmacology and Therapeutics, Ruxmaniben Deepchand Gardi Medical College, Ujjain, Ujjain, IND

**Keywords:** anticholinergic burden, elderly, online tools, polypharmacy, potentially inappropriate prescriptions

## Abstract

The elderly population differs from adults because they have numerous comorbid conditions and pharmacokinetics and pharmacodynamics profiles. The study aimed to detect inappropriate prescriptions in elderly patients using online tools in a tertiary care teaching hospital in Central India. This study was carried out in elderly inpatients who were prescribed more than five drugs to determine the inappropriate medications. For this, online tools such as MedStopper, Deprescribing.org and Anticholinergic Burden Calculator were used. Out of 291 drugs prescribed, 28 (10%) were identified as inappropriate using these online tools. Proton pump inhibitors were the most common potentially inappropriate prescriptions (PIPs) (73%) detected using MedStopper and Deprescribing.org. There was a total of 15 drugs identified as having high anticholinergic risk by the Anticholinergic Burden software. Of which, furosemide, alprazolam and digoxin were identified exclusively by this online software with appropriate substantiating evidence. This study provides insight into how to remain cautious while prescribing drugs to elderly patients and stimulates physicians to utilize the available tools in routine practice. By adequate compilation of many such Indian studies on the elderly population, it will be possible to develop guidelines for prescribing in the elderly Indian population.

## Introduction

The ageing population is a growing concern, particularly in India, where the proportion of elderly individuals is increasing rapidly, from 5.6% in 1961 to an estimated 12.4% by 2026 [1]. The elderly differ significantly from the younger population due to a higher prevalence of chronic and multiple diseases, disabilities, pharmacokinetic and pharmacodynamic changes, and social and behavioural shifts. This demographic is particularly susceptible to multimorbidities, necessitating the use of multiple medications to manage concurrent conditions. Consequently, elderly patients are the largest consumers of medications, leading to significant concerns about geriatric polypharmacy [2].

The extensive use of medications in this age group elevates the risk of adverse drug reactions (ADRs) and drug-drug interactions. These ADRs can result in a prescribing cascade, where additional medications are prescribed to address adverse effects, thereby increasing the overall pill burden [3]. The prevalence of inappropriate medication use among the elderly ranges from 11.5% to 62.5%, with the risk of inappropriate prescribing rising alongside polypharmacy. Inappropriate prescribing includes the use of medications without a clinical indication, medications where the risks outweigh the benefits, or when safer alternatives exist [4-6].

Since most of the randomized controlled trials exclude elderly populations, it is challenging to apply the results of these studies to older adults. Therefore, it is essential to use specific criteria and tools to guide medication prescribing in this demographic. While various offline tools, such as the STOPP/START criteria and Beers criteria [7], are available, there is a need for quick and accessible evidence-based online tools to facilitate appropriate prescribing, especially for busy clinicians [8].

Hence, this retrospective study utilized online tools such as MedStopper, Deprescribing.org, and Anticholinergic Burden Calculator to identify the potentially inappropriate prescriptions (PIPs) in elderly patients.

## Materials and methods

This study was carried out as a retrospective analysis of 295 case files of elderly patients admitted between 1st March 2018 and 29th February 2020 in the Departments of General Medicine, Cardiology and Nephrology after obtaining Institute Ethics Committee approval from All India Institute of Medical Sciences, Raipur, India with a study duration of one year. Since this was a retrospective study and the risk associated with it was less than minimal, the patients’ and physicians’ identities were not at all revealed at any stage during and after the study.

Aim of the study

To assess the appropriateness of prescription using online tools such as MedStopper (https://medstopper.com), Deprescribing.org (https://deprescribing.org) and Anticholinergic Burden Calculator (www.anticholinergicscales.es/) in elderly patients.

Objective

The objective of the study was to find out the usefulness of online tools such as MedStopper, Deprescribing.org and Anticholinergic Burden Calculator for detecting inappropriate prescriptions in elderly patients.

Inclusion criteria

The in-patient case files of patients aged 60 and above of either sex, admitted in the Departments of General Medicine, Cardiology and Nephrology who were prescribed more than five drugs were included in this study.

Exclusion criteria

The case files of elderly patients who were deceased or discharged within 48 hours of hospitalisation, discharged against medical advice, admitted for pure infective aetiology without any co-existing comorbid conditions, admitted in the critical care unit and with incomplete case files were excluded from this study.

Data collection

The data collected from the case files include the patient’s demographic details, diagnosis, clinical findings, and prescription chart in the form of the number, dose, route, duration and frequency of drugs prescribed. The number of case files in General Medicine, Cardiology and Nephrology were 242, 38 and 15, respectively. Every 10th case file was selected after applying inclusion and exclusion criteria. Each patient’s medication charts were evaluated for appropriateness in prescription using MedStopper, Deprescribing.org and Anticholinergic Burden Calculator.

Statistical analysis

Descriptive statistics in the form of proportions were used to represent the data. Data analysis was carried out with the help of Microsoft Excel (Version 16; Microsoft® Corp., Redmond, WA).

## Results

Demographic and prescription data

Upon analyzing the data of 295 elderly patients, the majority of the patients were males (63%) and the mean age was 66.10 ± 5.99 years. The average number of drugs per prescription was 12.17 ± 4.88. The total number of drugs prescribed was 291. Table 1 shows the demographic and prescription details of the included patients’ case files.

**Table 1:** Demographic and prescription details of the patients’ case files PIPs: potentially inappropriate prescriptions

S. no.	Characteristics	Observed data
1.	Gender	
	Male	186 (63%)
	Female	109 (37%)
2.	Age	
	60-69	214 (73%)
	70-79	64 (22%)
	>=80	17 (5%)
	Age (Mean ± SD)	66.10 ± 5.99 years
3.	Average number of drugs per prescription	12.17 ± 4.88
4.	Total number of drugs prescribed	291
5.	Total number of PIPs identified	28 (10%)

Potentially inappropriate medications (PIMs) identified using online tools

The total encounters of PIPs identified were 972 using online tools. MedStopper detected a greater number of PIPs. Table 2 depicts the number of encounters with PIPs as well as the drugs identified as PIMs without repetition, using online tools.

**Table 2:** Encounters of PIPs and number of drugs identified as PIMs using online tools

S. no.	Online tools to detect potentially inappropriate prescriptions (PIPs)	Encounters of PIPs, n (%)	Number of drugs identified as potentially inappropriate medications (PIMs), n (%)
1.	MedStopper	530 (26)	25 (43)
2.	Deprescribing.org	306 (15)	4 (7)
3.	Anticholinergic Burden Calculator	136 (7)	15 (26)

Drugs identified as PIMs using MedStopper

Table 3 illustrates the detailed description of each drug identified as PIMs using MedStopper with the recommendation provided in the criteria and the number of patients who were prescribed each drug. The most common PIPs were proton pump inhibitors followed by diuretics.

**Table 3:** Description of individual drug identified as PIM using MedStopper PIMs: potentially inappropriate medications; COPD: chronic obstructive pulmonary disease Note: Denominator - total number of patients, i.e., 295.

S. no.	Drugs identified as PIMs using MedStopper with the recommendation to reduce the dose if used daily for >3-4 weeks	Number of patients who were prescribed, n (%)
1.	Proton pump inhibitors	214 (73)
2.	Diuretics	126 (43)
3.	Benzodiazepines	48 (16)
4.	Sliding scale insulin	29 (10)
5.	First generation anti-histamines	26 (9)
6.	Tramadol	21 (7)
7.	Levetiracetam	12 (4)
8.	Nifedipine	10 (3)
9.	Amiodarone	7 (2)
10.	Prazosin	6 (2)
11.	Pregabalin	6 (2)
12.	Clonidine	3 (1)
13.	Carbamazepine	3 (1)
14.	Systemic corticosteroids for COPD	2 (0.6)
15.	Digoxin	2 (0.6)
16.	Diltiazem	2 (0.6)
17.	Antidepressants	2 (0.6)
18.	Amitriptyline	2 (0.6)
19.	Antipsychotic	2 (0.6)
20.	Metoclopramide	2 (0.6)
21.	Paracetamol	1 (0.3)
22.	Z-drugs	1 (0.3)
23.	Fluoxetine	1 (0.3)
24.	Oxcarbazepine	1 (0.3)
25.	Dicycloverine	1 (0.3)

Drugs identified as PIMs using Deprescribing.org

Table 4 illustrates the detailed description of each drug identified as PIMs using Deprescribing.org with the recommendation provided in the criteria and the number of patients who were prescribed each drug. The most common PIPs were proton pump inhibitors followed by antihyperglycaemic drugs.

**Table 4:** Description of individual drug identified as PIMs using Deprescribing.org PIMs: potentially inappropriate medications Note: Denominator - total number of patients, i.e., 295.

S. no.	Drugs identified as PIMs using Deprescribing.org	Recommendation	Number of patients who were prescribed, n (%)
1.	Proton pump inhibitors	Dose reduction or stop and use on-demand/non-pharmacological approach	214 (73)
2.	Anti-hyperglycemic (sliding scale insulin, long-acting sulfonylureas)	Engage the patient and/or caregivers to watch for signs and symptoms of hypoglycemia, adverse effects of drugs; Set blood glucose and A1C targets, implement drug changes-switch to an agent with lower risk of hypoglycemia, Reduction of doses of renally eliminated drugs (eg: metformin, sitagliptin), Monitor daily for 1-2 weeks after change for signs of hyperglycemia/hypoglycemia/adverse effects/Increased frequency of blood glucose monitoring if needed.	42 (14)
3.	Benzodiazepine	Taper slowly and stop; To be avoided in older adults. Oxazepam and Lorazepam can be preferred.	48 (16)
4.	Antipsychotic	The risks outweigh the benefits, consider psychiatric consultation. Watch for adverse withdrawal symptoms.	2 (0.6)

Anticholinergic burden calculator

A total of 15 drugs were identified as having high anticholinergic risk which furosemide, alprazolam and digoxin were detected as having high anticholinergic risk exclusively by this web-based software, whereas they were not present in other offline tools upon scrutiny.

Drugs labelled as having high anticholinergic risk

Table 5 shows the list of drugs detected using the web tool as having high anticholinergic properties according to different scales used.

**Table 5:** List of drugs identified as having high anticholinergic risk/burden

S. no.	List of drugs with high anticholinergic burden
1.	Furosemide
2.	Ipratropium bromide
3.	Chlorpheniramine maleate
4.	Alprazolam
5.	Digoxin
6.	Hydroxyzine
7.	Clonidine
8.	Amitriptyline
9.	Hyoscine
10.	Quetiapine
11.	Trihexyphenidyl
12.	Metoclopramide
13.	Dicyclomine
14.	Atropine
15.	Glycopyrrolate

## Discussion

In recent years, there has been a significant increase in the ageing population. Elderly individuals face unique challenges compared to younger adults, including a higher incidence of chronic and multiple diseases, disabilities, social and behavioural changes, and alterations in pharmacokinetics and pharmacodynamics. To manage these multiple health conditions, the elderly often undergo polypharmacy, which involves the use of numerous medications. This practice can lead to dangerous outcomes such as ADRs, non-compliance, and harmful drug-drug interactions. The risk of ADRs is particularly high in the elderly due to metabolic changes and decreased drug clearance [9].

A critical issue related to polypharmacy in the elderly is the prevalence of inappropriate medication prescriptions. Such prescribing practices can exacerbate the complexity of managing multiple health conditions and introduce additional health risks. This issue is especially relevant in the Indian context, where specific attention and action are needed. Most research and guidelines on geriatric medication use are based on studies conducted in Western countries, highlighting the need for guidelines that are specifically tailored to the Indian elderly population [10].

The majority of the patients in our study were males (63%) within 60-69 years (73%). In a study conducted by Salwe KJ et al. [11] and Karandikar YS et al., [12], 62% (in both the studies) were males, almost similar to our study. In the study by Salwe KJ et al. [11], the mean age of the patients was 71.6 ± 6.51 years, while in our study, the mean age is 66.10 ± 5.99 years. Regarding prescription data, the majority of patients were prescribed 6-10 drugs, with an average of 12.17 ± 4.88 drugs per prescription in our study. These findings are consistent with those reported by Salwe KJ et al. [11] and Chandrasekhar D et al. [13].

In our study, many patients were admitted for cardiac diseases (31%), such as mitral stenosis, mitral regurgitation, non-ST-elevation myocardial infarction (NSTEMI), and STEMI, followed by neurological diseases (22%) like cerebrovascular accidents and cerebral venous sinus thrombosis. Similarly, Roux B et al. [14] found that 36.4% of their patients had cardiac comorbidities. Buda V et al. [15] reported cardiovascular conditions as the leading cause of hospitalization, particularly essential hypertension (82.35%), followed by neurological disorders like dementia (3%). These patterns align with our findings. Conversely, Grace A et al. [16] found that falls (27.3%) and respiratory illnesses (15.8%) were the most common causes in an emergency department setting, with chronic kidney disease being the least common (7%). This difference is attributed to the different study settings.

MedStopper (https://medstopper.com/) identified a total of 530 (26%) PIPs, 482 in General Medicine, 25 in Cardiology and 23 in Nephrology, contributing to 25 (43%) drugs. MedStopper also utilizes STOPP and Beers criteria for analysis where a separate column is specified for the suggestions from Beers/STOPP criteria. Since there are no studies which have utilized this tool, there is a lack of data for comparison of results. MedStopper identified 25 drugs as PIPs. The most common ones were proton pump inhibitors (73%) followed by diuretics (43%). There were no studies utilizing MedStopper to compare our results.

Deprescribing.org, a web-based software detected a greater number of PIPs, i.e., a total of 306 (15%) encounters, 273 in General Medicine, 20 in Cardiology and 13 in Nephrology departments with a total of four (7%) categories of drugs such as proton pump inhibitors, anti-hyperglycaemic drugs, benzodiazepines and antipsychotics. One more category that is present in the Deprescribing software is the cholinesterase inhibitors and memantine, for which no drug was detected in our study. While constructing web-based software for Deprescribing, the concerned authorities use multiple resource materials, of which offline tools such as STOPP/START criteria, Beers criteria etc., play a major role (https://deprescribing.org/what-is-deprescribing/). Deprescribing.org categorizes the queries as whether to reduce, stop or continue the category of medication based on the subset of questions for each option. There are also options for monitoring therapy guidelines when reducing, stopping or switching medication and guidelines for the management of signs and symptoms while deprescribing [17]. Using Deprescribing.org, PIPs belonging to four categories of drugs were identified such as proton pump inhibitors (73%), anti-hyperglycaemic (14%) and benzodiazepine receptor agonists (17%). The recommendation for proton pump inhibitors is "Dose reduction or stop and use on-demand/Non-pharmacological approach/OTC antacids." The signs of hypoglycaemia have to be monitored while prescribing anti-hyperglycaemic. The suggestion for benzodiazepine receptor agonists is "to avoid in elderly due to the risk of falls and fractures." In a study conducted by Urzal J et al. [18], the most commonly detected PIMs using Deprescribing.org were proton pump inhibitors (36%) followed by anti-hyperglycemic drugs (17%), which is in concordance with our study.

Anticholinergic Burden Calculator (https://www.anticholinergicscales.es/calculate) is a web-based tool to detect medications with anticholinergic properties and categorise the burden as high, medium and low severity according to different scales. "Anticholinergic burden" is defined as the cumulative effect of taking one or more drugs that are capable of developing anticholinergic adverse effects. Peripheral manifestations may occur such as urinary retention, constipation, and decreased secretions, amongst others and central manifestations such as delirium and cognitive and functional disorders that are more troublesome in elderly patients. This online tool is different from the previous ones in that this tool does not detect all the PIPs, but detects only those drugs with anticholinergic properties. This tool detected a total of 136 (7%) encounters of medications as having high anticholinergic risk/burden, 123 in General Medicine, seven in Cardiology and six in Nephrology Departments, contributing to 15 (26%) drugs. It is less in comparison with a study conducted by Tristancho-Pérez Á et al. [19] where there were a total of 80 anticholinergic drugs. The Anticholinergic Burden Calculator tool uses 10 different scales based on evidence to calculate the burden. In our study, we have refined the results by including only those drugs that had "High" anticholinergic properties in any of the 10 scales used rather than compiling the sum of the scores in each scale. If the scores from each scale were considered, most of the prescriptions had a high anticholinergic burden. The reason is that this online software included drugs like "furosemide" which is not widely known as having anticholinergic properties. However, based on the proper evidence from the study, which is mentioned in the reference to that particular scale, such drugs are identified as having anticholinergic properties. "Furosemide" has "low" anticholinergic risk according to the Anticholinergic Cognitive Burden Scale (ACB), Chew’s scale and Anticholinergic Activity Scale (AAS), whereas the scale Anticholinergic Burden Classification (ABC) specifies it as having high anticholinergic burden. Among the identified drugs as having high anticholinergic burden, furosemide, alprazolam and digoxin are the drugs, identified only by the online software, whereas these drugs were not present in other offline tools upon scrutiny. A study by Chew ML et al. on anticholinergic medications commonly used in older age groups was published in the *Journal of the American Geriatrics Society*. The anticholinergic risk was demonstrated at higher concentrations of tested drug levels. The results were interpreted as above-average C(max) values in patients who received higher doses of furosemide and digoxin [20]. Raei K et al. [21] in their study observed alprazolam 11.3% according to the Drug Burden Index (DBI) scale and Anticholinergic Drug Scale (ADS) as one of the anticholinergic medications prescribed to elderly patients. The most commonly encountered drugs with anticholinergic risk in our study were furosemide (38% patients), ipratropium bromide (11% patients) and chlorpheniramine maleate (8% patients). Among the 15 main anticholinergic drugs prescribed to elderly patients with benign prostatic hyperplasia, furosemide was prescribed in 294 (7.8%) patients and chlorpheniramine in 144 (3.8%) patients in a study by Lopez-Rodriguez JA et al. [22] This aspect of this online software can be considered superior to other common offline tools since it brings the not-so-commonly studied drugs with anticholinergic risk into light with sufficient evidence mentioned on their website. While transferring the use of this software into clinical practice, the physician can get more precise information regarding the potential risks before prescribing to vulnerable populations like elderly patients so that it can be altered with some other medication. This method can be utilized for patients who are not in need of emergency medical care. In emergency situations, treating physicians will not get ample time to weigh the benefits-risk ratio and where limited drugs are available to save the lives of patients. This web-based tool will also aid in academic projects, for example, the anticholinergic risk of uncommon drugs mentioned in this software can be explored exclusively in either a specific subgroup or a wider population. More studies utilizing this online tool for anticholinergic burden will be beneficial to assess the authenticity of the results obtained.

This study highlights common medication-related problems in the elderly, i.e., the prescription of inappropriate medications. These findings underscore the need to enhance medication reconciliation strategies for elderly patients. Currently, there are no specific prescription guidelines tailored to the geriatric population in the Indian context. By addressing this gap, our study will contribute to the development of prescription guidelines for the elderly Indian population.

This pioneering study utilized multiple online tools to assess prescriptions in elderly patients and thoroughly analyze the results. The usage of different tools provides a better insight into this unexplored topic. As a retrospective study, it did not provide immediate feedback to the treating physicians. However, the findings will be helpful for the treating physicians in improving the quality of patient care. The study findings were communicated to the physicians after the completion.

## Conclusions

More PIPs were identified with MedStopper. The most frequently encountered drugs as PIMs with MedStopper were proton pump inhibitors followed by diuretics. Using Deprescribing.org, PIPs belonging to four categories of drugs were identified such as proton pump inhibitors, anti-hyperglycaemic and benzodiazepine receptor agonists. The most commonly encountered drugs with anticholinergic risk in our study were furosemide, ipratropium bromide and chlorpheniramine maleate. Among the drugs detected as having high anticholinergic burden, furosemide, alprazolam, and digoxin were identified only by the Anticholinergic burden calculator tool based on sufficient evidence. Compiling numerous such studies will be instrumental in developing prescription guidelines tailored for the Indian elderly population as well as adding value to prescribers while prescribing drugs to the elderly.
